# Spermatophyta Molecular Clock: Time Drift and Recent Acceleration

**DOI:** 10.1002/pei3.70084

**Published:** 2025-09-18

**Authors:** Soichi Osozawa

**Affiliations:** ^1^ Institute of Geology and Paleontology Faculty of Science, Tohoku University Sendai Japan

**Keywords:** base substitution rate, BEAST v1.10.4, C_4_ plants, calibration, glacier period, radiation

## Abstract

Angiospermae radiation is widely recognized as a mid‐Cretaceous event, but the adaptive radiation of *Asarum* and *Viola* as spring ephemerals also occurred during the Quaternary. To better understand the evolution of Angiospermae through geological time, a robust and well‐calibrated timetree for Spermatophyta was constructed. The Angiospermae topology was aligned with the APG (Angiosperm Phylogeny Group) system, although node dates tend to be overestimated in recent mega‐trees compared to those in this study. An exponential increase in the base substitution rate in recent geologic time was also revealed through the use of an alternative dating function in BEAST (Bayesian Evolutionary Analysis Sampling Trees) v1.10.4, followed by a presumed increase in the mid‐Cretaceous. These events are thought to correspond to the Angiospermae radiations at the species level during the Quaternary and at the order level during the mid‐Cretaceous. One possible cause of the recent increase in substitution rates and subsequent radiations, including those of *Asarum* and *Viola*, could be the proliferation of C_4_ grasses, a reduction in atmospheric CO_2_, and the onset of the Quaternary glacial period. Plant evolution has drastically altered Earth's environments, which, in turn, have influenced evolutionary processes. The mid‐Cretaceous event may have been driven by co‐radiation with herbivorous beetles, although beetles also include species with non‐herbivorous habits.

## Graphical abstract

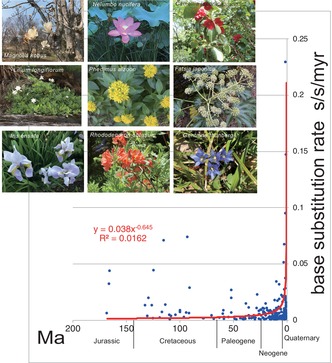

## 1 Introduction

The current phylogenetic dating analyses show an increasing base substitution (or mutation) rate in recent times, which may result from adaptation to severe environmental changes, particularly glaciations. Plant evolution has been closely linked to Earth's physical environment, especially atmospheric composition, through photosynthesis, as highlighted below.

Cyanobacteria (also known as blue‐green algae, though they are not true algae) are Gram‐negative bacteria (prokaryotes), and their growth products are stromatolites. Living cyanobacteria and stromatolites are found in Shark Bay, Australia, and fossil stromatolites have been identified in Australian strata dating from 3.5 to 3.3 billion years ago (Ga; Schopf and Packer 1987). The chloroplast is believed to have originated from cyanobacteria and was incorporated into the descendant eukaryotes (Gray 1992; Martin and Rujan 2004). Cyanobacteria were the first organisms to produce oxygen through photosynthesis, transforming the oxygen‐poor atmosphere into an oxygen‐rich one in an event known as the Great Oxidation Event (2.4–2.1 Ga; Lyons et al. 2014). The increased concentration of oxygen in both the atmosphere and oceans led to the deposition of banded iron formations, which now represent most of the world's iron reserves. This event may also have contributed to a decrease in atmospheric methane and carbon dioxide (CO_2_) levels, diminishing the greenhouse effect and potentially triggering the Huronian glaciation around 2.3–2.2 Ga (Kopp et al. 2005). In this way, cyanobacteria played a crucial role in altering the physical environments and biological ecosystems of ancient Earth, particularly through the extinction of anaerobic organisms.

The Snowball Earth event around 650 million years ago (Ma) during the Cryogenian may have triggered the Cambrian Explosion, including the emergence of the Ediacaran fauna between 635 and 541 Ma (Pu 2016). However, the role of glaciation in this process remains debated and is not yet definitively established.

Land plants likely originated from cyanobacteria or their descendants and conduct photosynthesis in chloroplasts (plastids) acquired through endosymbiosis with cyanobacteria. During the Carboniferous period (358.9–298.9 Ma), atmospheric oxygen levels increased dramatically, while carbon dioxide levels decreased, largely due to the photosynthetic activity of tree ferns. This process resulted in the sequestration of carbon in coal deposits and had a significant impact on the Earth's climate (c.f., Wilson et al. 2017). The reduction in the greenhouse effect contributed to the Karoo glaciation (late Paleozoic ice age; Montañez and Poulsen 2013). Tree ferns played a profound role in shaping the Earth's environment. Osozawa and Nel (2024) also investigated this ice age using an approach similar to that of the present study.

The formation of the Antarctic Circumpolar Current isolated the Antarctic continent and may have triggered the development of the present Antarctic ice sheet. This event is dated to the initiation of Scotia Plate spreading after chron C11n (around 30 Ma; Riley et al. 2019), which led to the formation of the Drake Passage, as discussed in Osozawa (2023). Another, and possibly the primary, factor contributing to the Quaternary glaciation may have been a decrease in atmospheric carbon dioxide levels. These levels declined from the greenhouse conditions of the Cretaceous to the Paleogene and Neogene, and into the Quaternary (Pearson and Palmer 2000; c.f., Bartoli et al. 2011).

It has been proposed that the reduction of atmospheric CO_2_ during the Quaternary may have been driven by plant photosynthesis, in a manner analogous to the process observed during the Carboniferous period (Osozawa and Wakabayashi 2022; Neoptera, Osozawa 2023; Mammalia, and Osozawa and Nel 2024; Paleoptera). If this hypothesis is correct, atmospheric composition and the Earth's environment would have been significantly influenced by Spermatophyta, and most likely Angiospermae, through photosynthesis in chloroplasts.

**FIGURE 1:**
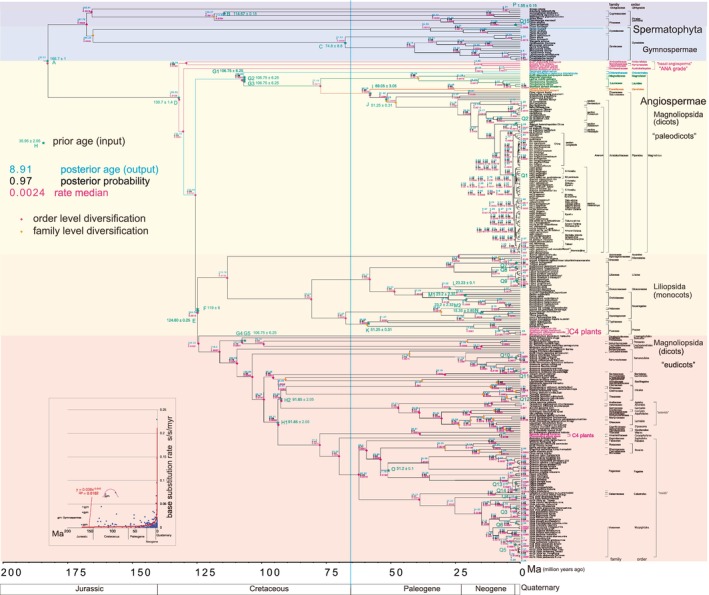
Bayesian inference tree of Spermatophyta based on the combined ITS (694 bp), matK (833 bp), and rbcL (544 bp) sequences (not concatenated) constructed using BEAST v1.10.4. For Gymnospermae, family and order names are based on Christenhusz et al. (2011) and Yonekura and Murata (2013). For Angiospermae, family and order names are based on the APG III system (APG III 2009), shown alongside each species (Yonekura and Murata 2013). Inset (bottom left): Base substitution rate (median rate shown at each node) vs. age (posterior age shown at each node) diagram. The heavy red approximate curve with the exponential equation, quantitatively and statistically defensible, was drawn using an Excel function, with the curve intersection at 0.038. Because the exponential curve does not fit well with the Cretaceous‐Jurassic data, the lighter curve was drawn manually. The older‐aged Gymnospermae plot is marked “gym”.

A key goal of botany is to correlate botanical evolutionary events with the timeline of Earth's history (Wilf and Escapa 2015). The present paper aims to achieve this objective by examining the ultimate interactions between plants and the environment. To this end, Spermatophyta Bayesian inference (BI) trees were constructed as an initial trial using the latest version of BEAST v1.10.4, with genes, including chloroplastic genes, incorporated. The BI tree was simultaneously dated using a multipoint calibration function within BEAST v1.10.4, providing an accurate depiction of Spermatophyta (including chloroplast) evolution from the Jurassic to the Quaternary, spanning 182 million years (Figure 1). *Ginkgo biloba* (Ginkgoidae) and representative species of *Pinales* (conifers) and *Cycadales* were included for Gymnospermae, as well as *Amborella trichopoda* for the oldest species in the “ANA grade” (APG IV 2016) of Angiospermae. In addition, the rest of the ANA grade (Nymphaeales and Austrobaileyales), along with Chloranthales and Magnoliidae (Magnoliales, Laurales, Canellales, and Piperales), which are referred to as “paleodicots” by Leitch et al. (1998; see also APG II 2003), were also included.

Angiosperms were diversified during the Cretaceous, and their adaptive radiation may have been linked to an increased base substitution rate (Magallón et al. 2015). It was also suggested that, while significant morphological and functional diversity in angiosperms has deep evolutionary roots, the high species richness observed in extant angiosperms was likely developed more recently, during the Quaternary. A correlation between higher substitution rates and species richness and diversification in the Proteaceae was demonstrated by Duchêne and Bromham (2013) ( *Platanus orientalis* , Proteales, eudicots, are included in my analyses; Figure 1). Furthermore, it was shown by Sun et al. (2020), Zuntini et al. (2024), Dimitrov et al. (2023), and Benton et al. (2022) that diversification rates increased over the last 15 million years, coinciding with a period of global cooling.

Within Angiospermae, plants with the C_4_ photosynthetic pathway, known as C_4_ plants, dominate today's tropical savannahs and temperate grasslands, accounting for approximately 30% of global terrestrial carbon fixation (Osborne and Beerling 2006). A major evolutionary innovation from C_3_ to C_4_ photosynthesis occurred between 33 and 23 Ma (Sage 2004; Christin et al. 2011). Although the CO_2_‐concentrating pump in C_4_ plants enhances photosynthetic efficiency in warm climates with low atmospheric CO_2_ concentrations (Sage 2004; Osborne and Beerling 2006), an alternative interpretation is proposed here: that the rise of C_4_ photosynthesis contributed to the decline in atmospheric CO_2_ by improving carbon fixation efficiency. In the present analyses, Poaceae (Poales; monocots) and Amaranthaceae (Caryophyllales; dicots) were included (Figure 1).

The issue of accelerated base substitution rates is revisited through calibration across both younger ages, such as Quaternary species‐level divergences (e.g., spring ephemerals like *Asarum* and *Viola*; Osozawa and Nackejima 2025), and older ages (e.g., *Ginkgo biloba* and *Amborella trichopod*a). A similar approach to that outlined in Osozawa and Wakabayashi (2022), Osozawa (2023), and Osozawa and Nel (2024) is followed in the present study. According to these studies, Quaternary calibrations were identified as playing a key role in the construction of a reliable dated phylogenetic tree. It is proposed here that the species diversity of Angiospermae in recent times (Soltis et al. 2019; Benton et al. 2022) can largely be attributed to the recent increase in base substitution rates, which were driven by adaptive radiation in response to climatic changes. The primary factors behind this shift were identified as C_4_ photosynthesis, the decline in atmospheric CO_2_, and global cooling.

## 2 Materials and Methods

The present dated tree was constructed using a total of 291 taxa and 2071 bp of sequence data, employing BEAST v1.10.4 (Bayesian Evolutionary Analysis Sampling Trees; Suchard et al. 2018). The sample size and genome length are relatively small compared to those used in recent phylogenetic studies, as shown in Table 1. However, as noted in Osozawa and Nel (2024), the following observation also applies to the present study:

The recent trend in phylogenetic studies may be leading to larger trees and genome sizes, potentially resulting in more precise and detailed phylogenetic trees. However, while increased tree and genome sizes can enhance the robustness of the topology, they do not guarantee accurate dating. In other words, tree and genome sizes alone cannot calibrate the phylogenetic tree and are therefore insufficient for dating purposes.

**TABLE 1:** Compilation of the dated phylogenetic tree of Spermatophyta.

References	Target	Tree siza	Genome size	Application	References	Calibration point	Dating	Topology	Radiation
Bremer (2000)	Monocots	91	rbcL	×	×	8	Inflated	Concordant	Early Cretaceous‐Paleogene
Qiu et al. (2010)	Angiosperm (mostly)	380	5100 bp	RAxML 7.0.4	Stamatakis et al. (2008)	×	×	Concordant	×
Smith et al. (2010)	Angiosperm (mostly)	154	rbcL etc.	BEAST (Ver. 1.4.7)	Drummond and Rambaut (2007)	33	Extremely inflated	Concordant	Triassic‐Paleogene
Soltis et al. (2011)	Angiosperm (mostly)	640	25,260 bp	RAxML 7.0.4	Stamatakis et al. (2008)	×	×	Concordant	×
Nagalingum et al. (2011)	Cycadales	199	rbcL matK PHYP	BEAST v1.5	Drummond and Rambaut (2007)	4	Concordant	Concordant	Neogene
Condamine et al. (2015)	Cycadales	237	rbcL matK PHYP	BEAST v1.8 (+ maximum age)	Drummond et al. (2012)	6	Extremely inflated	Concordant	Permian‐Neogene
Magallón et al. (2015)	Angiosperm (mostly)	799	9089 bp	BEAST v1.7.5 (+ maximum age)	Drummond et al. (2012)	137	Inflated	Concordant	Early Cretaceous‐Paleogene
Givnish et al. (2018)	Monocots	567	83,478 bp	BEAST v2.4.7	Bouckaert et al. (2014)	17	Inflated	Concordant	Early Cretaceous‐Paleogene
Foster et al. (2017)	Angiosperm (mostly)	195	76 protein‐coding genes	MCMCTree in PAML (4.8)	Yang et al. (2007)	37	Extremely inflated	Concordant	Triassic‐Cretaceous
Ran et al. (2018)	Gymnosperm (mostly)	38	1,296,042 bp	MCMCTree in PAML (4.9a)	Yang et al. (2007)	14	Extremely inflated	Concordant	Carboniferou‐Paleogene
Barba‐Montoya et al. (2018)	Angiosperm (mostly)	644	75,030 bp	MCMCTree in PAML (4.8)	Yang et al. (2007)	52	Inflated	Concordant	Early Cretaceous‐Neogene
Smith and Brown (2018)	Angiosperm (mostly)	79,881	75,030 bp	TreePL (+ maximum age)	Smith and O'Meara (2012)	62	Inflated	Concordant	Early Cretaceous‐Neogene
Li et al. (2019)	Angiosperm (mostly)	2539	82,286 bp	TreePL (+ maximum age)	Smith and O'Meara (2012)	62	Extremely inflated	Concordant	Triassic‐Cretaceous
OTPTI (2019)	Viridiplantae	1153	410 nuclear gene	ASTRAL	Zhang et al. (2018)	×	×	Concordant	×
Lubna et al. (2021)	Gymnosperm (mostly)	167	128,080 bp	BEAST v2.4.7	Bouckaert et al. (2014)	13	Extremely inflated	Concordant	Devonian‐Neogene
Li et al. (2021)	Angiosperm (mostly)	4660	Plastomes	RAxML v8.2.12	Stamatakis (2014)	×	×	Concordant	×
Zuntini et al. (2024)	Angiosperm	7923	353 nuclear genes	TreePL (+ maximum age)	Smith and O'Meara (2012)	200	Extremely inflated	Concordant	Late Jurassic‐Paleogene
Present paper	Angiosperm (mostly)	291	2071 bp	BEAST v1.10.4	Suchard et al. (2018)	25	Original	Original	Early Cretaceous‐Quaternary

The current sample size and sequence length are sufficient to provide adequate resolution for analysis using BEAST v1.10.4.

### 2.1 Taxon Sampling

*Amborella trichopoda*, originating from New Caledonia, was provided by the Koishikawa Botanical Garden, University of Tokyo. Some Angiospermae specimens, for which sequence data were unavailable in DDBJ/GenBank, were collected from the Experimental Station for Medical Plant Studies, Graduate School of Pharmaceutical Sciences, Tohoku University. Newly collected specimens of *Asarum*, *Viola*, and other spring ephemeral species endemic to Japan, the Ryukyu Islands, and Taiwan for the present study are described in detail in Osozawa and Nackejima (2025), including in Table 1 of that study.

### 2.2 DNA Extraction, Polymerase Chain Reaction Amplification, and Sequence Alignment

Refer to section 2.4, “DNA Extraction and Polymerase Chain Reaction Amplification,” in Osozawa and Nackejima (2025), along with the accompanying Table 1, for the sequence data also used in the present study. The chloroplastic maturase K (matK) gene, the chloroplastic ribulose‐1,5‐bisphosphate carboxylase/oxygenase large subunit (rbcL) gene, and the nuclear ribosomal internal transcribed spacer (ITS) region were selected for the present analyses. These genes are relatively short in base pair length, but they provided sufficient resolution for the phylogenetic analyses (Osozawa and Nackejima 2025). The evolutionary history of the chloroplast can also be inferred using these genetic markers.

### 2.3 Phylogenetic Analyses Associated With Fossil and Geological Event Calibrations by BEAST v1.10.4

A Bayesian Inference (BI) tree (Figure 1) was constructed using BEAST v1.10.4. For additional details on constructing the base substitution rate (“rate median” shown at each node in FigTree) and node age (“Node age”) diagram (Figure 1 inset), as well as Figure S1 (relative rate analysis using the MEGA11 function, Tamura et al. 2021) and Figure S2 (calibration date analysis), see Osozawa and Nackejima (2025), Osozawa (2023), and Osozawa and Nel (2024).

The calibration points and dates are outlined below:

In a literature survey on fossil calibrations, it was identified that Smith et al. (2010) employed geologically reasonable and geochronologically reliable calibration dates. Representative fossil calibrations provided by Magallón et al. (2015; Supporting Information Methods S1: Fossil‐Derived Calibrations) and Barba‐Montoya et al. (2018; NOTES S1: Justification of Fossil Calibrations) were also considered. These calibrations, along with other rigorously evaluated examples summarized in Table 2, were utilized in the present study.

**TABLE 2:** Fossil and geological event calibrations. Geological event calibrations at 1.55 ± 0.15 Ma were considered based on Osozawa and Nackejima (2025).

Calibration point	Fossil	Family	Order	Ingroup clade	Formation	System	Stage	tMRCA (Ma)	Method	Paleontological references	Geological references
A	† *Ginkgoxylon gruetii*	Ginkgoaceae	Ginkgoales	*Ginkgo biloba* (stem)	Tiaojishan Formation	Jurassic	Bathonian	166.7 ± 1	Ar‐Ar dating	Jiang et al. (2016)	Chang et al. (2009, 2014)
B	† *Araucaria grandifolia*	Cupressaceae	Pinales	Cupressaceae	Punta del Barco Formation	Cretaceous	Aptian	114.67 ± 0.18	U–Pb dating	Del Fueyo and Archangelsky (2002)	Césari et al. (2011)
B1 (not applied)	† *Protophyllocladoxylon*	Podocarpaceae	Pinales	Pinales	La Meseta Formation	Paleogene	Eocene Priabonian	35.95 ± 2.05	Biostratigraphy	Pujana et al. (2014)	Cocozza and Clarke (1992)
C	† *Wintucycas*	Zamiaceae	Cycadales	Zamiaceae	Allen Formation	Cretaceous	Campanian‐Maastrichtian	74.8 ± 8.8	Biostratigraphy	Martinez et al. (2012)	Martinez et al. (2012)
C1 (not applied)	† *Ceratozamia floersheimensis*	Zamiaceae	Cycadales	Zamiaceae	Most Formation	Paleogene	Oligocene Rupelian	32.9 ± 0.9	Biostratigraphy	Kvacek (2014)	Kovar‐Eder (2016)
C2 (not applied)	† *Dioon praespinulosum*	Zamiaceae	Cycadales	*Dioon* (stem)	Kootznahoo Formation	Paleogene	Paleocene‐Miocene	43.22 ± 22.78	Correlation	Erdei et al. (2012)	Brew et al. (1984)
D1	† *Archaefructus liaoningensis*	Archaefructaceae		*Amborella trichopoda* (stem)	Yixian Formation	Cretaceous	Hauterivian	130.7 ± 1.4	Ar‐Ar dating	Sun et al. (2002, 2011)	He et al. (2006)
D2	† *Archaefructus liaoningensis*	Archaefructaceae		Angiospermae	Yixian Formation	Cretaceous	Hauterivian	130.7 ± 1.4	Ar‐Ar dating	Sun et al. (2002, 2011)	He et al. (2006)
E	† *Leefructus mirus*	Ranunculaceae	Ranunculales	Eudicots (stem)	Yixian Formation	Cretaceous	Hauterivian	124.60 ± 0.25	Ar‐Ar dating	Sun et al. (2011)	Yang et al. (2007)
F	Pollens			Monocots	Portugal continental rise	Cretaceous	Aptian	119 ± 6	Palynology	Hochuli et al. (2006)	Hochuli et al. (2006)
G1	† *Scutifolium jordanicum*	Cabombaceae	Nymphaeales	Nymphaeales (stem)	Mahis flora	Cretaceous	Albian	106.75 ± 6.25	Correlation	Taylor et al. (2008)	Taylor et al. (2008)
G2		Chloranthaceae	Chloranthales	Chloranthales (stem)	Potomac Group	Cretaceous	Albian	106.75 ± 6.25	Correlation	Crane et al. (1994)	Crane et al. (1994)
G3		Magnoliaceae+	Magnoliales+	Magnoliidae (stem)	Potomac Group	Cretaceous	Albian	106.75 ± 6.25	Correlation	Crane et al. (1994)	Crane et al. (1994)
G4		Buxaceae	Buxuales	Buxuales (stem)	Potomac Group	Cretaceous	Albian	106.75 ± 6.25	Correlation	Crane et al. (1994)	Crane et al. (1994)
G5		Nelumbonaceae	Proteales	Proteales (stem)	Potomac Group	Cretaceous	Albian	106.75 ± 6.25	Correlation	Crane et al. (1994)	Crane et al. (1994)
H1		Crassulaceae+	Saxifragales	Saxifragales (stem)	Raritan Formation	Cretaceous	Turonian	91.85 ± 2.05	Correlation	Hermsen et al. (2003)	Hermsen et al. (2003)
H2		Ericaceae+	Ericales	Ericales (stem)	Raritan Formation	Cretaceous	Turonian	91.85 ± 2.05	Correlation	Hermsen et al. (2003)	Hermsen et al. (2003)
I	† *Piper margaritae*	Piperaceae	Piperales	Piperales(stem)	Guaduas Formation	Cretaceous	Maastrihitian	69.05 ± 3.05	Correlation	Martinez et al. (2015)	Martinez et al. (2015)
J	† *Aristolochia mortua*	Aristolochiaceae	Aristolochiaceae	Piperales	Green River Formation	Paleogene	Eocene Ypresian	51.25 ± 0.31	Ar‐Ar dating	Grande (1984)	Smith et al. (2003)
J1 (not applied)	† *Aristolochia austriaca*	Aristolochiaceae	Aristolochiaceae	Piperales	Hollabrunn‐Mistelbach Formation	Neogene	Miocene Tortonian	9.61 ± 2.19	Correlation	Meller (2014)	Matenco and Radivojevic (2012)
K	† *Typha lesquereuxi*	Typhaceae	Poales	Typhaceae (stem)	Green River Formation	Paleogene	Eocene Ypresian	51.25 ± 0.31	Ar‐Ar dating	Grande (1984)	Smith et al. (2003)
L	† *Dioscorea wilkinii*	Dioscoreaceae	Dioscoreales	Dioscoreaceae	Guang River flora	Paleogene	Oligocene Chatian	27.23 ± 0.1	U–Pb dating	Pan et al. (2014)	Pan et al. (2014)
M1	† *Earina ouldenensis*	Orchidaceae	Asparagales	*Earina*	Foulden Hills diatomit	Paleogene	Oligocene Chatian	23.2 ± 0.232	Ar‐Ar dating	Conran et al. (2009)	Lindqvist and Lee (2009)
M2	† *Dendrobium winikaphyllum*	Orchidaceae	Asparagales	*Dendrobium*	Foulden Hills diatomit	Paleogene	Oligocene Chatian	23.2 ± 0.232	Ar‐Ar dating	Conran et al. (2009)	Lindqvist and Lee (2009)
N	† *Protoyucca shadishii*	Asparagaceae	Asparagales	*Yucca* (stem)	Virgin Valley Formation	Neogene	Miocene Langhian	15.35 ± 0.85	Ar‐Ar dating	Tidwell and Parker (1990)	Perkins et al. (1998)
O	† *Fagus langevinii*	Fagaceae	Fagales	Fagales	Klondike Mountain Formation	Paleogene	Eocene Ypresian	51.2 ± 0.1	U–Pb dating	Manchester and Dillhoff (2004)	Brew et al. (1984)
P	*Pinus luchuensis*	Pinaceae	Pinales	*Pinus luchuensis*+	Guga Formation	Quaternary	Pleistocene Calabrian	1.55 ± 0.15	Biostratigraphy	Osozawa and Watanabe (2012)	Osozawa et al. (2012)
Q1	Geological event	Aristolochiaceae	Piperales	*Heterotropa*	Ryukyu+	Quaternary	Pleistocene Calabrian	1.55 ± 0.15	Biostratigraphy	Osozawa et al. (2012)	Osozawa et al. (2012)
Q2	Geological event	Aristolochiaceae	Piperales	*Asiasarum*	Ryukyu+	Quaternary	Pleistocene Calabrian	1.55 ± 0.15	Biostratigraphy	Osozawa et al. (2012)	Osozawa et al. (2012)
Q3	Geological event	Violaceae	Malpighiales	*Viola okinawensis*+	Ryukyu+	Quaternary	Pleistocene Calabrian	1.55 ± 0.15	Biostratigraphy	Osozawa et al. (2012)	Osozawa et al. (2012)
Q4	Geological event	Violaceae	Malpighiales	*Viola amamiana*+	Ryukyu	Quaternary	Pleistocene Calabrian	1.55 ± 0.15	Biostratigraphy	Osozawa et al. (2012)	Osozawa et al. (2012)
Q5	Geological event	Violaceae	Malpighiales	*Viola yedoensis*+	Ryukyu+	Quaternary	Pleistocene Calabrian	1.55 ± 0.15	Biostratigraphy	Osozawa et al. (2012)	Osozawa et al. (2012)
Q6	Geological event	Violaceae	Malpighiales	*Viola orientalis*+	Japan	Quaternary	Pleistocene Calabrian	1.55 ± 0.15	Biostratigraphy	Osozawa et al. (2012)	Osozawa et al. (2012)
Q7	Geological event	Liliaceae	Liliales	*Trillium*	Japan	Quaternary	Pleistocene Calabrian	1.55 ± 0.15	Biostratigraphy	Osozawa et al. (2012)	Osozawa et al. (2012)
Q8	Geological event	Liliaceae	Liliales	*Erythronium*	Japan	Quaternary	Pleistocene Calabrian	1.55 ± 0.15	Biostratigraphy	Osozawa et al. (2012)	Osozawa et al. (2012)
Q9	Geological event	Liliaceae	Liliales	*Lilium alexandrae*+	Japan	Quaternary	Pleistocene Calabrian	1.55 ± 0.15	Biostratigraphy	Osozawa et al. (2012)	Osozawa et al. (2012)
Q10	Geological event	Ranunculaceae	anunculales	*Adonis*	Japan	Quaternary	Pleistocene Calabrian	1.55 ± 0.15	Biostratigraphy	Osozawa et al. (2012)	Osozawa et al. (2012)
Q11	Geological event	Ranunculaceae	anunculales	*Anemone*	Japan	Quaternary	Pleistocene Calabrian	1.55 ± 0.15	Biostratigraphy	Osozawa et al. (2012)	Osozawa et al. (2012)
Q12	Geological event	Theaceae	Ericales	*Camellia japonica*	Japan	Quaternary	Pleistocene Calabrian	1.55 ± 0.15	Biostratigraphy	Osozawa et al. (2012)	Osozawa et al. (2012)
Q13	Geological event	Fagaceae	Fagales	*Fagus crenata*+	Japan	Quaternary	Pleistocene Calabrian	1.55 ± 0.15	Biostratigraphy	Osozawa et al. (2012)	Osozawa et al. (2012)
Q14	Geological event	Fagaceae	Fagales	*Fagus japonica*+	Japan	Quaternary	Pleistocene Calabrian	1.55 ± 0.15	Biostratigraphy	Osozawa et al. (2012)	Osozawa et al. (2012)
Q15	Geological event	Pinaceae	Pinales	*Pinus luchuensis*+	Ryukyu+	Quaternary	Pleistocene Calabrian	1.55 ± 0.15	Biostratigraphy	Osozawa et al. (2012)	Osozawa et al. (2012)

*Note*: † denotes extinct taxa.

Abbreviations: Ma, million years ago; tMRCA, time of the most recent common ancestor.

Some calibrations are based on radioisotopic dating of fossil‐bearing strata, while others rely on biostratigraphy correlated with specific ages or stages on the geologic time scale. Absolute age ranges for these stages are typically derived from radioisotopic dating of associated strata in key global localities (Wilf and Escapa 2015). The geologic time scale has been standardized by the International Commission on Stratigraphy (ICS) (www.stratigraphy.org). The latest version, v2024/12, is accompanied by an explanatory paper detailing its development by Cohen et al. (2013).

In addition to the geological event calibration points Q1–Q15 at 1.55 ± 0.15 Ma (Osozawa and Nackejima 2025), fossil calibration points A–P (with P corresponding to Q15) are shown in Figure 1 and detailed in Table 2.

Calibration point A: *Ginkgo biloba* , often referred to as a living fossil and an extant genus, is represented by fossil wood reported from Liaoning Province, northern China (Jiang et al. 2016). Chang et al. (2009, 2014) provided Ar‐Ar ages of 160.7 ± 0.4 Ma and 166.7 ± 1.0 Ma for these strata. I adopted the latter as the prior input and stem age.

Calibration point B: *Araucaria grandifolia* was reported from the Punta del Barco Formation (Baqueró Group), Santa Cruz Province, Argentina (Del Fueyo and Archangelsky 2002). These strata were dated using the U–Pb method to 114.67 ± 0.18 Ma (Césari et al. 2011). Calibration point B2 (not applied, as it is much younger than B): Conifer fossil woods were reported from the Eocene La Meseta Formation, Antarctica (Pujana et al. 2014). Marine microplanktonic fossils suggest a Priabonian age of 35.95 ± 2.05 Ma for these rocks (Cocozza and Clarke 1992).

Calibration point C: The extinct *Wintucycas* (Zamiineae) was reported from the Allen Formation, Argentina (Martinez et al. 2012). This formation, which contains dinosaur fossils, represents the first Atlantic transgression (Armas and Sánchez 2015) and is considered Late Cretaceous (Campanian to Maastrichtian, 74.8 ± 8.8 Ma). Calibration point C1 (not applied): Extant and extinct *Ceratozamia* species were reported from European basinal strata (Kvacek 2014). These correspond to calcareous nannoplankton zone NP23 (Oligocene, 31.8 ± 2.2 Ma) and planktonic foraminifera zone P18 (32.9 ± 0.9 Ma; Kovar‐Eder 2016). Calibration point C2 (not applied): Fossil *Dioon praespinulosum* was reported from the Kootznahoo Formation, Alaska (Erdei et al. 2012). However, these strata span an extensive range, including the entire Paleogene and early Miocene (Brew et al. 1984), making precise dating difficult. Fossil localities of modern cycads from Australia are similarly constrained by loose geochronologic data. Note: Extinct species such as *Nilssonia* (Nilssoniales) and *Antarcticycas* cannot be used as calibration dates for the extant orders analyzed in this study (c.f., Coiro et al. 2023).

Calibration Point D1: *Archaefructus*, an extinct genus from the Jehol Biota in northeast China, is the earliest known genus of Angiosperms. The lower *Archaefructus* fossil horizon within the Yixian Formation (Sun et al. 2002, 2011) was dated using the Ar‐Ar method applied to intercalated silicic tuff, yielding a date of 130.7 ± 1.4 Ma (He et al. 2006). This date was used as the crown age for the entire group of Angiosperms. Calibration Point D2: Fossils of *Amborella* have not been found, but this species represents the oldest lineage of Angiosperms in the APG system (APG IV. 2016). The stem age for Amborella was also set at 130.7 ± 1.4 Ma.

Calibration Point E: *Leefructus mirus*, also from the Jehol Biota, belongs to a basal eudicot family, the Ranunculaceae (Ranunculales), and provides the earliest fossil record of tricolpate pollen (eudicot pollen; Sun et al. 2011; c.f., Walker and Walker 1984; Donoghue and Doyle 1989; Bao et al. 2019; Smith and Beaulieu 2024). The fossil horizon within the middle Yixian Formation (Sun et al. 2011) was dated using the Ar‐Ar method applied to sanidine in intercalated silicic tuff, yielding a date of 124.60 ± 0.25 Ma (Yang et al. 2007).

Calibration Point F: Monocolpate pollen data from the Early Cretaceous continental sequences of western Portugal revealed that monocot radiation occurred during Aptian time (119 ± 6 Ma) (Hochuli et al. 2006). It was suggested by the authors that this radiation preceded the radiation of dicots by at least 10 million years. However, this conclusion is not adopted here, as it conflicts with the calibrations presented in points D and E, as well as with the APG system (APG IV 2016).

Dicot fossil data relevant to the fossil calibrations in this study were reviewed by Smith et al. (2010). Calibration G: Nymphaeales (G1, ANA grade; paleodicots) fossils were found in Albian strata of Jordan, dated to 106.75 ± 6.25 Ma (Taylor et al. 2008). Fossils of Chloranthales (G2, paleodicots), Magnoliidae (Magnoliales + Laurales + Piperales; G3, paleodicots), Buxuales (G4, eudicots), and Proteales (G5, eudicots) were recovered from the Albian Potomac Group, also dated to 106.75 ± 6.25 Ma (Crane et al. 1994; Crane and Herendeen 1996).

Calibration Point H: Saxifragales (H1, eudicots) and Ericales (H2, eudicots) fossils have been reported from the Turonian Raritan Formation, USA, dated to 91.85 ± 2.05 Ma (Hermsen et al. 2003; Nixon and Crepet 1993).

Calibration Point I: Fossil leaves of Piper (Piperales, paleodicots) have been reported from Maastrichtian coal seams in Colombia, South America (Martinez et al. 2015). Therefore, the time of the most recent common ancestor (MRCA) of all species in Piperales, including *Piper*, *Houttuynia*, *Aristolochia*, and *Asarum*, was 69.05 ± 3.05 Ma (Figure 1). This calibration point was also used in Osozawa and Nackejima (2025).

Calibration Point J: An Eocene *Aristolochia* fossil was reported from the Green River Formation (Grande 1984), with an Ar‐Ar age of 51.25 ± 0.31 Ma reported for these rocks (Smith et al. 2003). This calibration point was also used in Osozawa and Nackejima (2025). J1 (not applied): A fossil of *Aristolochia* (Piperales) was reported from the western Pannonian Basin, Austria (Meller 2014), and was estimated to be of Tortonian age (9.61 ± 2.19 Ma; Miocene) according to Matenco and Radivojevic (2012).

Iles et al. (2015) reviewed monocot fossil data relevant to the fossil calibrations used in this study (Figure 1, Table 2).

Calibration Point K: A *Typha* fossil was reported from the Green River Formation (Grande 1984), with an associated Ar‐Ar age of 51.25 ± 0.31 Ma (Smith et al. 2003).

Calibration Point L: A *Dioscorea* fossil was discovered in strata in Ethiopia, which yielded a U–Pb radiometric age of 27.23 ± 0.1 Ma (Pan et al. 2014). This calibration point was also used in Osozawa and Nackejima (2025).

Calibration Points M1 and M2: *Fossils of Earina and Dendrobium* were reported from strata in New Zealand (Conran et al. 2009), with an Ar‐Ar age of 23.2 Ma assigned to the deposits (error margins not reported; Lindqvist and Lee 2009).

Calibration Point N: *Protoyucca* was found in strata in Nevada (Tidwell and Parker 1990), and silicic tuff intercalated with these layers was dated by the Ar‐Ar method to 15.35 ± 0.85 Ma (Perkins et al. 1998).

Calibration Point O: As compiled by Momohara and Ito (2023), *Fagus langevinii* was reported from the Klondike Mountain Formation in Washington (Manchester and Dillhoff 2004), with a U–Pb age estimated at 51.2 ± 0.1 Ma (Rubino et al. 2021). This calibration point was also used in Osozawa and Nackejima (2025).

Calibration point P (= Q16): *Pinus luchuensis* are endemic to the Ryukyu Islands. A cone fossil of *P. luchuensis* was found in the 1.55 Ma Guga Formation on Okinawa Island (Osozawa and Watanabe 2011), and the aforementioned geologic event calibration (Q16) is consistent with the fossil calibration date. Fossils of *Cunninghamia lanceolata* (Pinidae), *Cerbera manghas* (Gentianales), *Schima wallichii* (Ericales), *Liquidambar formosana* (Saxifragales), *Caesalpinia crista* (Fabales), and *Quercus salicina* (Fagales) were also found in the Guga Formation (Osozawa and Watanabe 2011), and these species were included in the analyses.

The following calibration points, Q1–Q16, are based on the geological event calibration proposed by Osozawa et al. (2012) and subsequently applied by Osozawa and Nackejima (2025) with a calibration date of 1.55 ± 0.15 Ma. See Table 2 for further details.

## 3 Results

### 3.1 Spermatophyta Timetree and Divergence Dates (Figure 1)

The present timetree spans a range dating back approximately 180 Ma, with no evidence of mutation saturation observed (Figure S1). Posterior probabilities at each node in Figure 1 are either 1 or close to 0.

The crown age of Spermatophyta was estimated at 182.61 Ma, that of Gymnospermae at 167.75 Ma, and that of Angiospermae at 132.03 Ma. Gymnospermae is a sister to Angiospermae.

The Gymnospermae consists of Ginkgoales (165.61 Ma; *Ginkgo biloba* ), Pinales (crown age at 118.73 Ma; Cupressaceae and Pinaceae), and Cycadales (crown age at 67.63 Ma; Cycadaceae and Zamiaceae) clades.

Paleodicot is a paraphyletic group, not monophyletic. The ANA grade (Amborellales, Nymphaeales, Austrobaileyales), Chloranthales, Magnoliales and Laurales, and Canellales are all paraphyletic with respect to their successive descendants. Canellales is sister to Piperales, while Chloranthales and Magnoliidae are sister to the clade comprising monocots and eudicots.

In the monocot clade, Acorales ( *Acorus calamus* ) represents the oldest lineage and is sister to Alismatales. In the eudicot clade, Ceratophyllales ( *Ceratophyllum demersum* ) is the oldest lineage and is placed as sister to all other eudicots. Buxales is sister to Proteales, Sabiales is sister to Ranunculales, and Santalales is sister to Gunnerales. Ericales is sister to the “asterids,” while Vitales and Caryophyllales and Sapindales are sister to the “rosids.”

Order‐level diversification began at 167.75 Ma (crown age) for Gymnospermae, between 132.03 Ma (Amborellales) and 58.13 Ma for paleodicots, between 124.79 Ma and 23.81 Ma for monocots, and between 124.33 and 46.74 Ma for eudicots. Family‐level differentiation occurred between 165.24 and 118.73 Ma for Gymnospermae, between 51.42 and 39.24 Ma for paleodicots, between 58.95 and 8.26 Ma for monocots, and between 90.83 and 3.24 Ma for eudicots. Genus‐species level differentiation partly overlapped with, but generally followed, family‐level differentiation.

Poales of the monocots began genus‐level diversification at 58.95 Ma, with C_4_ Poales grasses starting their diversification at 8.34 Ma (stem: 17.95 Ma). C_4_ Amaranthaceae (Caryophyllales) of the eudicots began diversification at 7.99 Ma (stem: 38.91 Ma). The significance of C_4_ plant radiation is discussed in detail in the discussion section.

For details on spring ephemerals of *Asarum* (Aristolochiaceae; Piperales), *Viola* (Malpighiales; Rosids), *Lilium*, *Trillium*, and *Erythronium* (Liliaceae; Liliales), and other relatively recently diversified genera such as *Adonis*, *Anemone*, *Camellia*, and *Fagus*, see Osozawa and Nackejima (2025).

### 3.2 Exponential Increase in Base Substitution Rate

The red curve and the equation fitted to the data are shown in the inset of Figure 1. The data suggest that the base substitution rate has increased exponentially since around 15 Ma (Miocene, Neogene). The Cretaceous data points in the inset of Figure 1 deviate from the approximate curve, with these rates being relatively high, including the Jurassic rates for Gymnospermae (marked “gym” in the inset).

## 4 Discussion

### 4.1 Relatively Recent Radiation of Gymnospermae (Table 1)

Nagalingum et al. (2011) developed a timetree for Cycadales, which includes the Cycadaceae and Zamiaceae clades. The tree incorporated multiple species from specific genera and represented a species‐level phylogeny. Speciation events occurred relatively recently, since the late Miocene, and they concluded that Cycadales is a living fossil. Species‐level analyses are needed to construct a more precise dated tree; see the next section for details.

Ran et al. (2018) constructed a deep phylogeny of Gymnospermae, including Ginkgoales, Pinales, Gnetales, and Cycadales. Lubna et al. (2021) further expanded this phylogeny by incorporating Araucariales and Cupressales. Although the diversification dates for deeper nodes appear to be overestimated (see the next section), Lubna et al. (2021) also reported relatively recent species‐level diversification.

Condamine et al. (2015) suggested that using the Yule prior in BEAUti resulted in a Paleogene diversification for extant cycad genera, while using the birth‐death prior led to a Neogene diversification. The present paper employed the Yule prior (noting that the birth‐death prior produced a similarly dated tree) and estimated that genus‐level differentiation in Cycadaceae occurred after 34.65 Ma (Eocene) and in Zamiaceae after 67.63 Ma (Maastrichtian). However, species‐level diversification predominantly occurred within the Neogene (Figure 1), and their study overestimated the diversification dates (see below for a discussion of this discrepancy).

### 4.2 Solving the “Jurassic Angiosperm Gap”: Post Jurassic Angiospermae Radiation

Recent Angiosperm mega‐trees tend to overestimate the ages of older branch nodes (summarized in Table 1). A “Jurassic angiosperm gap,” as observed by Li et al. (2019), refers to the discrepancy where molecular dating estimates are much older than fossil dates (c.f., Sauquet et al. 2022; Smith and Beaulieu 2024). According to Li et al. (2019), the crown age of Angiospermae (i.e., the stem age of Amborellales) is placed in the Triassic, and even the crown ages of monocots and eudicots are dated to the Jurassic. The overestimation arises from the use of MCMCTree in PAML (4.8) and its associated calibration function, which requires the application of maximum and root ages. As pointed out by Osozawa (2023) and Osozawa and Nel (2024), this results in estimated ages that are consistently older than the corresponding fossil ages. TreePL (Smith and O'Meara 2012) and BEAST 2 (Bouckaert et al. 2014) exhibit a similar issue (see Table 1). For a discussion of the limitations of BEAST 2, see section 2.4: Non‐utilization of BEAST 2 and MrBayes in Osozawa (2023). However, the BEAST v2.4 software used by Givnish et al. (2018) and Lubna et al. (2021) is an older version (see Table 1), one or more generations behind those addressed in Osozawa (2023), which focuses on BEAST v2.5 and later, including the current BEAST v2.7. Updating to the latest version is essential, as outdated software can compromise the accuracy of divergence time estimates. In contrast, fossil calibration ages yield similar node ages in my BEAST v1.10.4 dating (Figure 1), and therefore, the present molecular dates align closely with fossil dates, without any age gap.

Another factor contributing to the inflation of estimated dates in previous studies (Table 1) is the lack of Quaternary calibration, with the exception of Nagalingum et al. (2011). This omission tends to increase node dates by ignoring recent rapid base substitution rates (as opposed to older, slower rates, which is a new finding in the present study) and the impact of slow time passage. For further details, see Figure S2, as well as similar figures 4 and 5 in Osozawa and Nel (2024).

Angiosperm diversification began in the early Cretaceous (NOT the Permian, Triassic, or Jurassic) and has since undergone extensive radiation, as shown in Figure 1. The crown age of Angiospermae was estimated to be within the Cretaceous, at 132.03 Ma, with monocots diverging at 113.18 Ma and eudicots at 124.33 Ma. After near‐contemporaneous differentiations, order‐level diversification continued until the Miocene, at 23.81 Ma. Family‐level diversification began at 90.83 Ma in the Cretaceous and continued until 3.24 Ma in the Neogene, followed by genus‐species level differentiation, including the extensive Quaternary radiation of spring ephemerals (Osozawa and Nackejima 2025).

### 4.3 Triggers for the Late Cenozoic and Middle Cretaceous Radiation and Increases in Base Substitution Rates

Species differentiation in Spermatophyta appears to be particularly pronounced during the Quaternary period (Figure 1), as noted by Magallón et al. (2015), Smith and Brown (2018), Soltis et al. (2019), and Benton et al. (2022). Sun et al. (2020) demonstrated that the net diversification rate of the rosids, as estimated using BAMM v2.5.0 (Bayesian Analysis of Macroevolutionary Mixtures; Rabosky 2014), increased dramatically over the past 15 million years, coinciding with a cooling climate (c.f., Givnish et al. 2018). Zuntini et al. (2024) further showed that the net diversification rate of angiosperms has also risen since the Miocene, a phenomenon known as the “Cenozoic diversification surge,” which is linked to global climatic cooling. Although Dimitrov et al. (2023) observed a minor recent decline, with a more prominent diversification surge during the Cretaceous, they also highlighted a significant increase in diversification over the past 15 million years during cooling. Notably, the number of living species has increased exponentially, along with a rise in the base substitution rate, as illustrated by the similar trend curves in figure 2b of Benton et al. (2022) and the inset in Figure 1. This differentiation, which appears to be linked to the rising base substitution rate (inset in Figure 1), is examined here, along with a discussion of potential environmental triggers for the pattern, including the influence of climatic change and the evolution of C_4_ grasses.

A possible trigger for the increasing base substitution rates during the Quaternary may have been the onset of glacial and interglacial cycles, along with the severe environmental changes associated with this period. These changes could have influenced the radiation of spring ephemerals such as *Heterotropa* and *Viola* (Osozawa and Nackejima 2025). Feedback from biological developments may also have played a role in triggering the onset of glaciations. The expansion of C_4_ Poales grasses may have contributed to the Quaternary glaciations (Boom et al. 2001), while the late Paleozoic glaciations (Montañez et al. 2016) may have been triggered by the development of terrestrial tree ferns, which formed thick coal layers (see Osozawa and Nel 2024). These processes likely increased carbon fixation, which in turn lowered atmospheric CO_2_ concentrations (Taira 2007).

C_4_ plants are highly efficient in CO_2_ fixation (Sage 2004), and C_4_ Poales grasses began diversifying around 8.34 Ma (stem: 17.95 Ma; Figure 1), while C_4_ Amaranthaceae diverged from 7.99 Ma (stem: 38.91 Ma; Figure 1) as noted above. The revolutionary shift from C_3_ to C_4_ photosynthesis occurred during the Oligocene (23–33.9 Ma) and continued after 14.5 Ma (Sage 2004; Christin et al. 2011). Carbon isotope ratios from mammalian fossil tooth enamel suggest that dietary incorporation of C_4_ plants began around 9.9 Ma in eastern Africa (Uno et al. 2011). Isotopic analysis of mammalian fossil teeth from the sub‐Himalayan Siwalik Group in Pakistan shows that C_4_ savannas replaced C_3_ forests and woodlands between 8.5 and 6.0 Ma (Badgley et al. 2008). Additionally, the global expansion of C_4_ grasses, including in North and South America, began in the late Miocene and has continued through the present, including during the current glacial–interglacial cycles (Cerling et al. 1997). Note that the rapidly increasing base substitution rates observed in the present analyses began around 15 Ma (Figure 1 inset), not at 2.58 Ma with the onset of the Quaternary, and the duration of this period leading up to the Quaternary ice age was expected.

Osozawa and Wakabayashi (2022) noted that “Food plants of *Derotettix mendosensis* are C4 dicots of Amaranthaceae (see figure 9 in Sage 2004) and Chenopodiaceae in degraded salt‐plain habitats in arid regions of central Argentina (Simon et al. 2019). These dicot fossils and C_4_ monocot fossils of Poales grass (Chloridoidae) were reported from the Eocene in Patagonia by Zucol et al. (2018), and the fossil horizon was dated by the Ar‐Ar method at 49.512 ± 0.019 Ma (Woodburne et al. 2014). The C_4_ photosynthetic pathway began at ca. 50 Ma in South America, earlier than elsewhere.”

Although the focus has been on terrestrial plants, marine diatom evolution may also be connected to the global event of increasing base substitution rates and diversifications. Rabosky and Sorhannus (2009) proposed that grassland expansion led to increased silica in the oceans, which in turn stimulated the diversification of marine diatoms with silica skeletons. According to Lazarus et al. (2014), “Over the last 15 million years, diatom diversity is strongly correlated with the oxygen isotope proxy record of global climate change, the global carbon isotope record, and estimated past atmospheric pCO_2_. These correlations suggest that diatoms have played an important role in shaping mid‐Miocene to Recent climate, particularly through their prominent role in the oceanic carbon pump.” A time‐calibrated phylogeny of diatoms by Nakov et al. (2018) showed an increase in net diversification rates since the middle Miocene. In Japan, diatomite has been deposited along the Japan Sea coast since around 15 Ma, during the middle Miocene (Yanagisawa 1999).

The fossil record indicates a dramatic increase in both phylogenetic diversity and ecological abundance of angiosperms around the middle Cretaceous (Friis et al. 2006; Benton et al. 2022). This event may be linked to an increase in the base substitution rate, likely occurring around 110 Ma (Figure 1 inset), which may mark the beginning of order‐level radiation in Angiospermae (Figure 1). Subsequently, angiosperms underwent extensive diversification at the family and species levels (Figure 1). Insect beetles also exhibit a peak in their diversifications around the middle Cretaceous (Farrell 1998; Hunt et al. 2007; McKenna et al. 2015; Gunter et al. 2016; Toussaint et al. 2016; Zhang et al. 2018). This increase may be associated, at least in part, with the co‐radiation of angiosperms as food plants for certain beetle species. Broad‐leaved angiosperms, particularly dicots, played a key role in driving a global ecological transformation that contributed to Cretaceous biodiversity (Jan de Boer et al. 2012).

## 5 Conclusion

The Miocene expansion of C_4_ grasses reduced atmospheric CO_2_ levels through more efficient photosynthesis. This, in turn, triggered the Quaternary glacial and interglacial cycles, which contributed to an increase in base substitution and mutation rates, facilitating the extensive radiation of both plants and animals during the Quaternary. Biological processes influenced the Earth's environment, which then provided feedback to the evolution of life.

## Conflicts of Interest

The author declares no conflicts of interest.

## Supporting information


**Figure S1:** Relative rate analysis using the MEGA11 function (Tamura et al. 2021)

**Figure S2:** Calibration date analysis (see Osozawa and Nackejima 2025; Osozawa 2023; Osozawa and Nel 2024)

## Data Availability

All relevant data are within the manuscript. The sequence data of ITS, matK, and rbcL are available in GenBank/DDBJ, and the accession numbers are in table 1 in Osozawa and Nackejima (2025). The following publication is currently out of stock; however, a PDF version is available upon request from the senior author: Osozawa S, Watanabe Y. 2011. Geology of Nago City and Kunigami District, northern and central Okinawa main island, with colored geological map sheet. Nago, Okinawa, Nago Museum, 208 p. (in Japanese with English abstract).
