# Supplementary figures and images for: Spermatophyta Molecular Clock: Time Drift and Recent Acceleration
Source: Plant Environ Interact. 2025 Sep 18;6(5):e70084. doi: 10.1002/pei3.70084 (PMC12444776; doi:10.1002/pei3.70084)

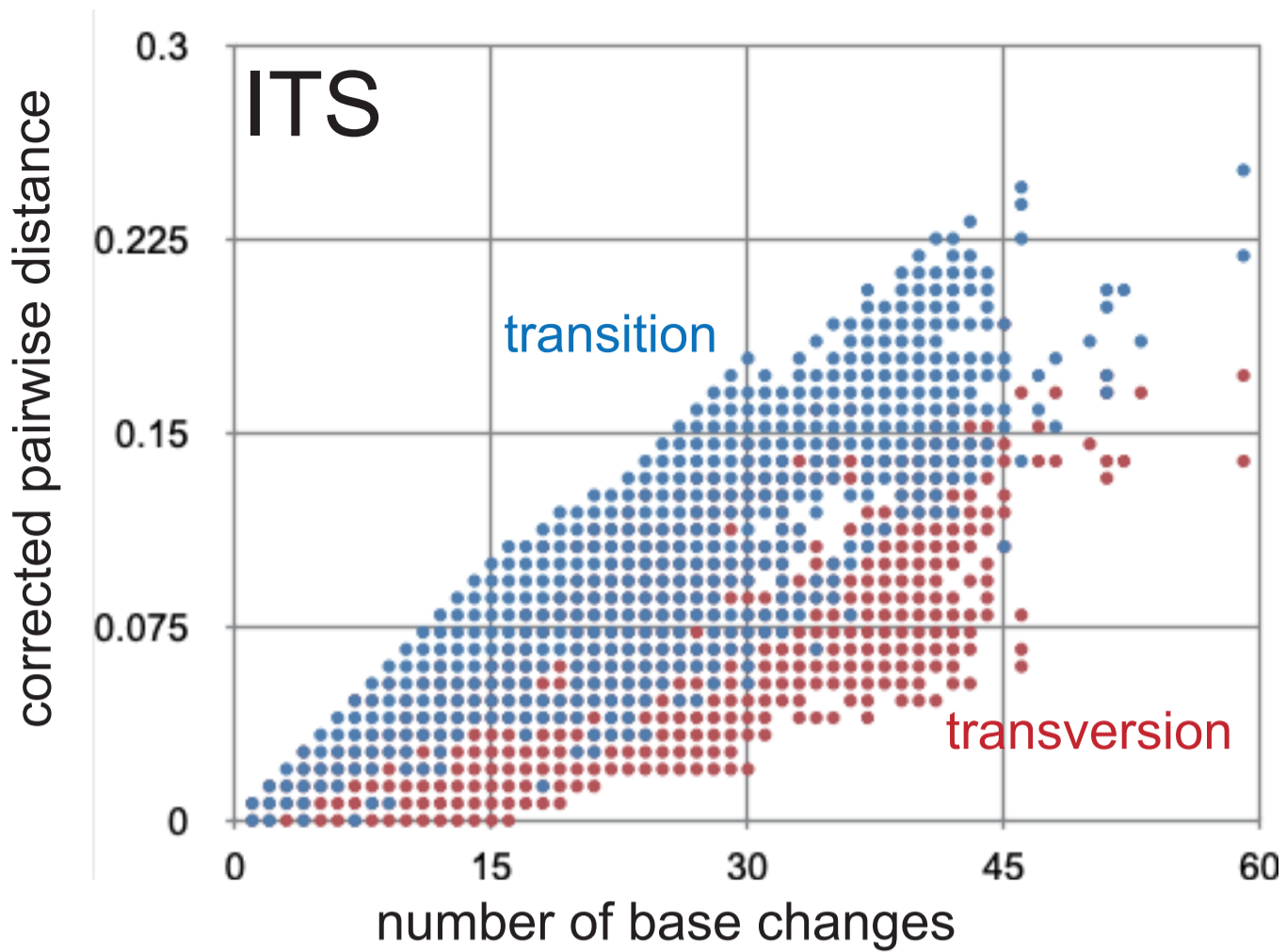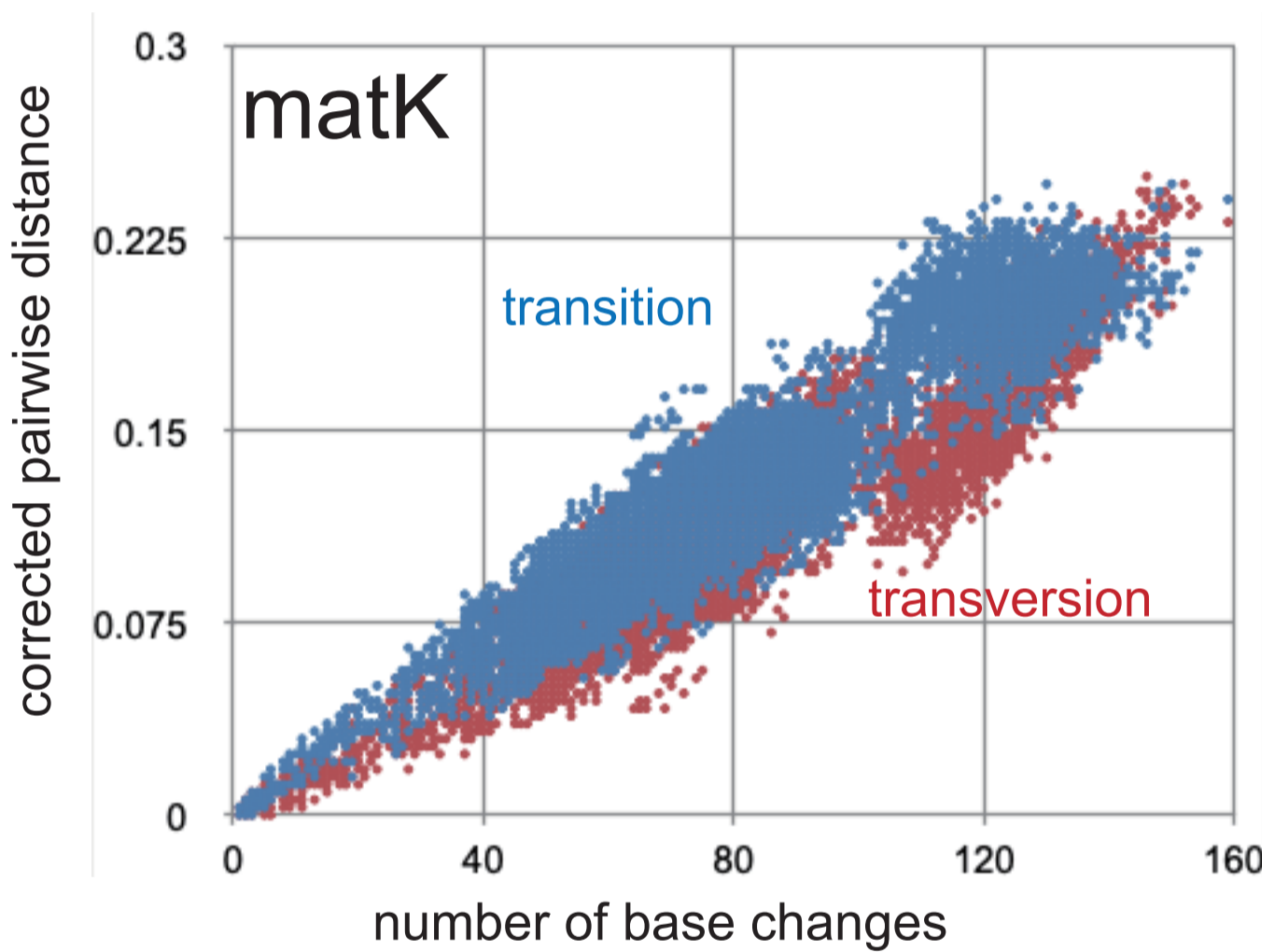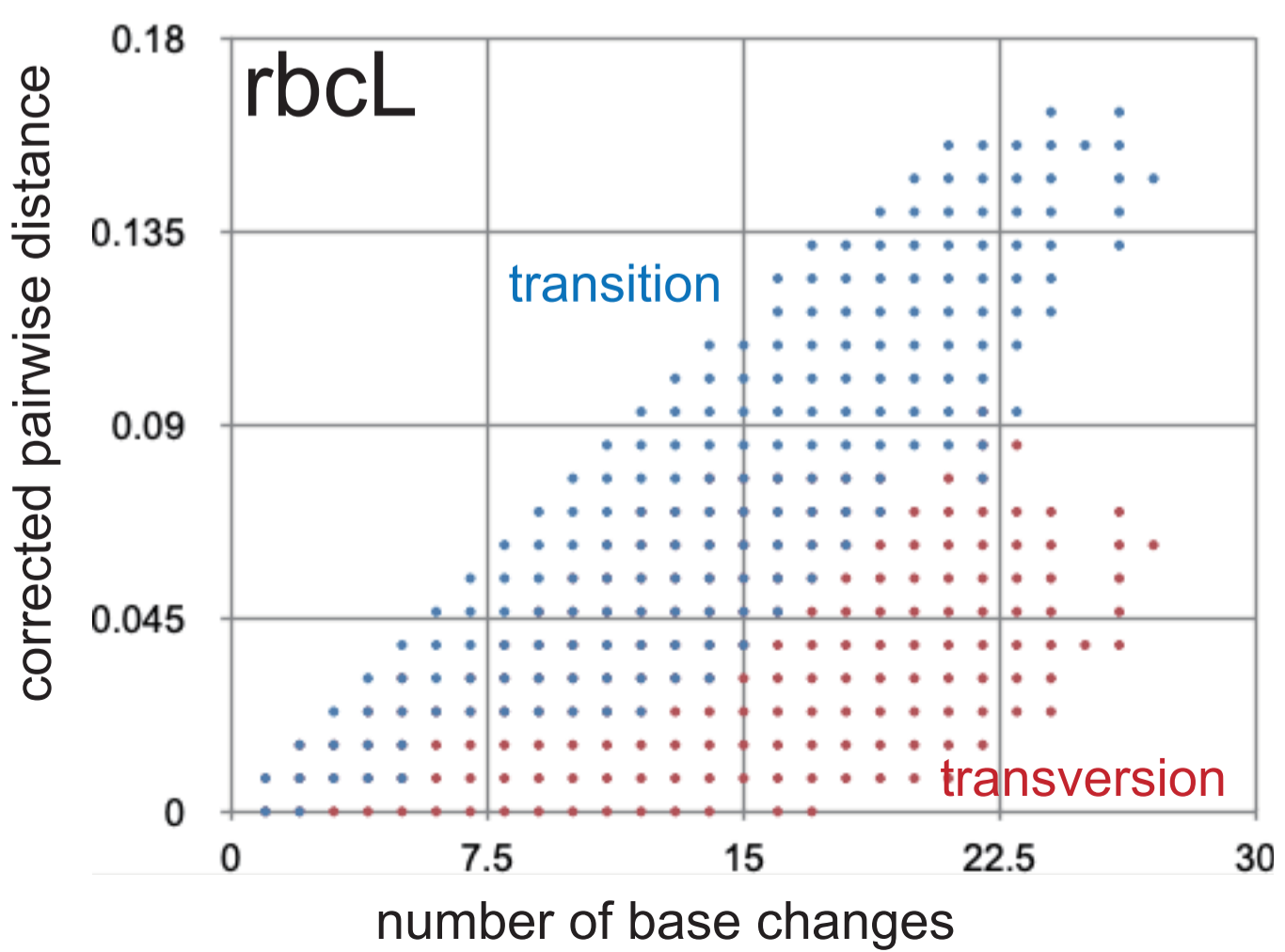

Supplement: Supplementary file 1 — Figure S1: Relative rate analysis using the MEGA11 function (Tamura et al. 2021) [file PEI3-6-e70084-s001.pdf]
