# Supplementary material for: Spermatophyta Molecular Clock: Time Drift and Recent Acceleration
Source: Plant Environ Interact. 2025 Sep 18;6(5):e70084. doi: 10.1002/pei3.70084 (PMC12444776; doi:10.1002/pei3.70084)
Supplement: Supplementary file 2 — Figure S2: Calibration date analysis (see Osozawa and Nackejima 2025; Osozawa 2023; Osozawa and Nel 2024) [file PEI3-6-e70084-s002.pdf]

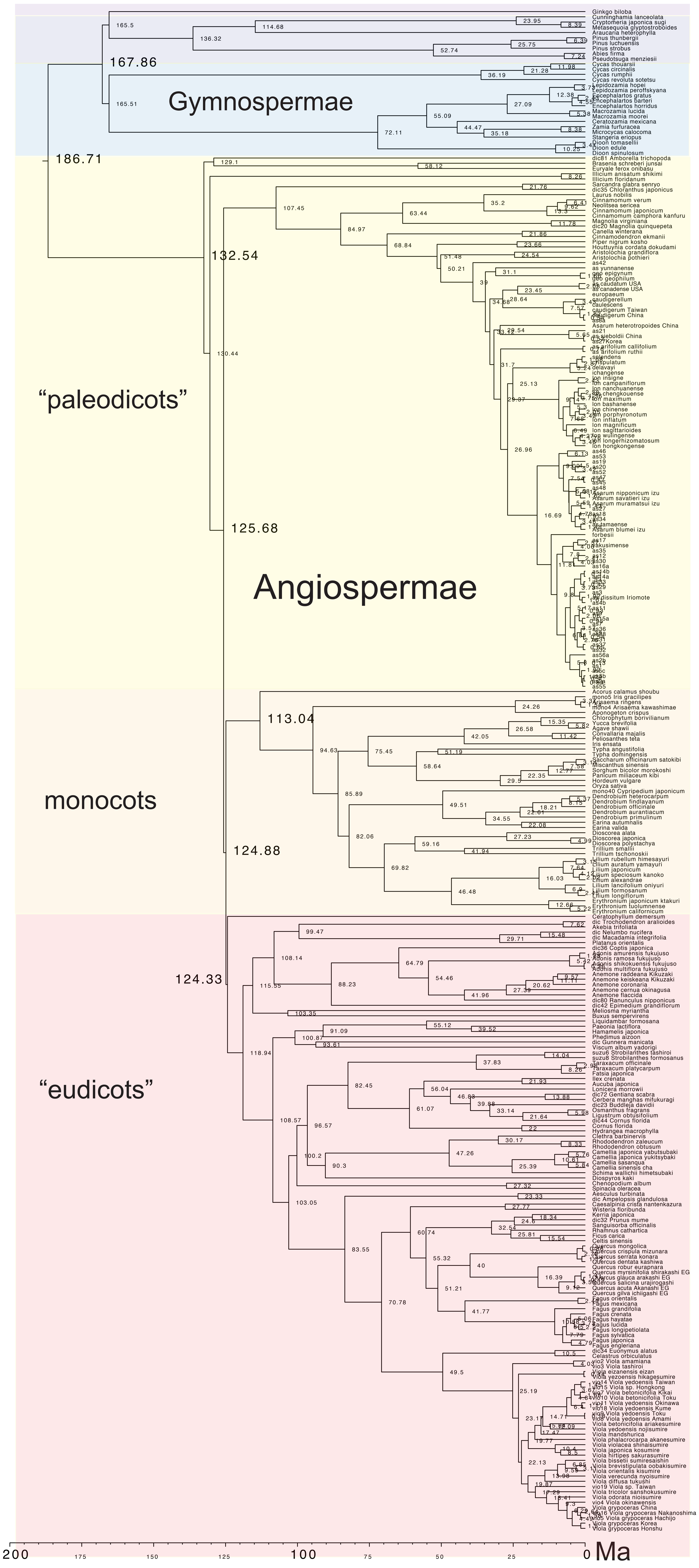


The timetree was calibrated exclusively using older dates, resulting in increased node ages.

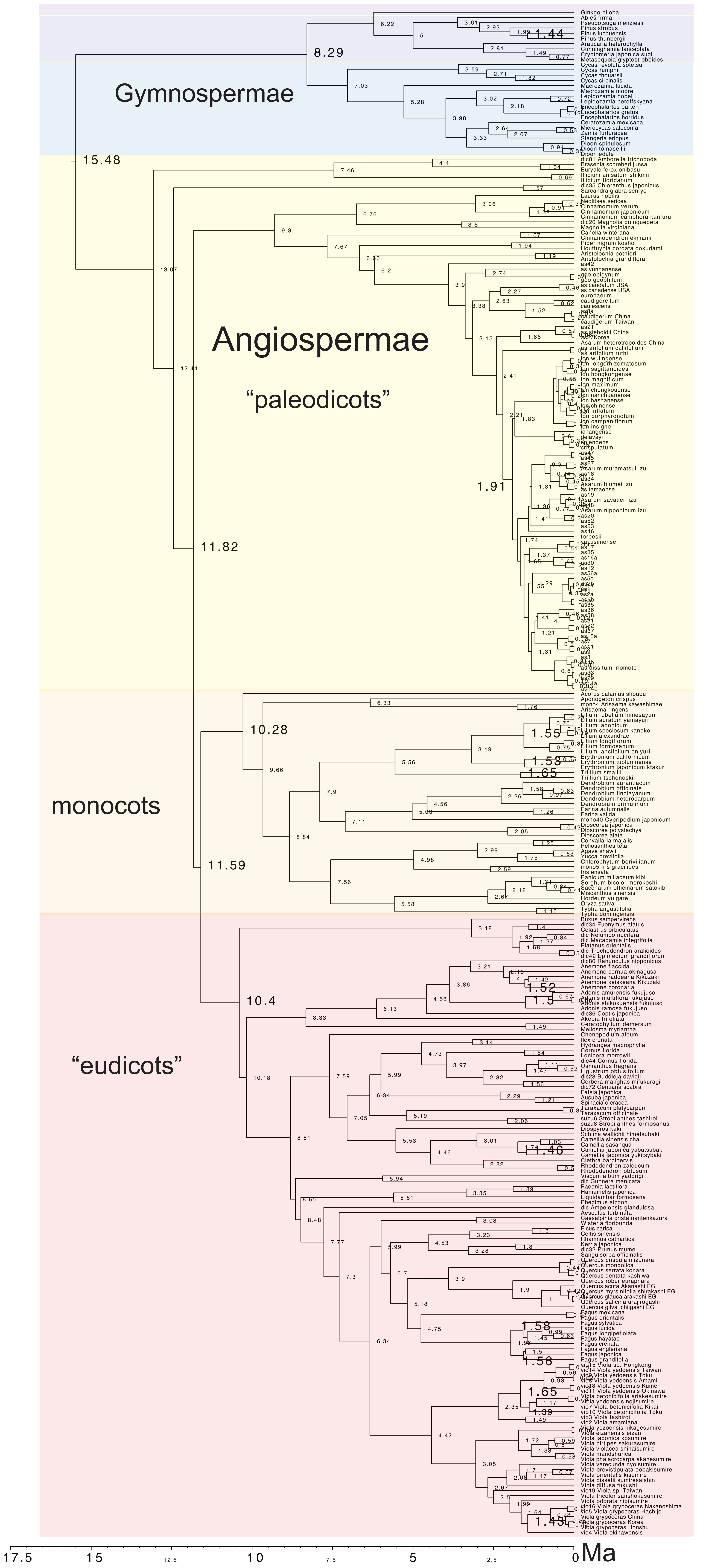


The timetree was calibrated exclusively using 1.55 Ma dates, resulting in significantly dreduced node ages.
